# A Single-Central, Randomized, Double-Blinded, Placebo-Controlled, Crossover Trial Protocol: A Clinical Effect Evaluation Study on the TCM Comprehensive Intervention Program for Chronic Heart Failure

**DOI:** 10.1155/2021/4577139

**Published:** 2021-12-28

**Authors:** Xingxing Li, Dong Li, Xiaoyun Cui, Kun Zhou, Jing Liu, Zongjing Fan, Jinjin Lu, Jie Wan, Rongkun Yan, Junyan Xia, Xing Li, Yang Wu, Qian Lin, Yan Li

**Affiliations:** ^1^Department of Graduate School, Beijing University of Chinese Medicine, Beijing 100029, China; ^2^Department of Cardiology, Dongfang Hospital, Beijing University of Chinese Medicine, Beijing 100078, China; ^3^Department of Cardiology, Dongzhimen Hospital, Beijing University of Chinese Medicine, Beijing 100700, China

## Abstract

**Background:**

It is known to all the doctors and patients that both the morality and incidence rate of chronic heart failure (CHF) are quite high among various heart diseases. Traditional Chinese medicine (TCM) comprehensive intervention becomes a rising prospective therapy for patients with CHF. Considering the efficacy of TCM, the study aims to test the safety as well as the validity of TCM comprehensive intervention in patients who are struggling with CHF.

**Methods:**

The study is an essentially randomized, single-central, placebo-controlled, double-blinded crossover trial. Eighty-two eligible subjects aged 18–75 years with CHF are supposed to be recruited. According to the subject plan, all the patients will be divided into group A and B. The patients in group A will receive oral Qishen Taohong granules (QTGs) combined with TCM external treatment and standard Western medicine for four weeks. After that, a 2-week washout would be proceeded; this group will be reallocated to another four weeks with oral placebo granules combined with TCM external treatment and standard Western medicine. In contrast, group B will perform the opposite protocol. The primary outcome conforms to the classification from the New York Heart Association (NYHA). Meanwhile, the secondary outcomes are echocardiogram, N-terminal pro-B-type natriuretic peptide (NT-proBNP), Chronic Heart Failure Quality of Life Scale of Integrated Chinese and Western Medicine (CHFQLS), TCM syndrome, symptom, sign, six-minute walk test (6MWT), Pittsburgh Sleep Quality Index (PSQI), Montreal cognitive assessment (MoCA), major adverse cardiovascular events (MACEs), and metabolomics. *Discussion*. Based on conventional treatments, TCM comprehensive intervention may further improve the patients' cardiac function and then enhance their quality of life. The results will provide high-quality evidence of TCM comprehensive intervention in treating CHF.

## 1. Introduction

Chronic heart failure (CHF) is the final form of cardiovascular diseases [1]. It could be fatal, disabling, and costly and has become an increasingly important global health problem [2]. CHF patients usually experience a malignant disease cycle: “hospitalization-improvement-discharge-rehospitalization” [3]. Epidemiological studies have shown that about 64.3 million people in the whole world are suffering different kinds of heart failure [4]. Recently, a meta-analysis was conducted with more than 1.5 million patients who have heart failure. The result shows that 1-, 2-, 5-, and 10-year survival rates, respectively, increase to 87%, 73%, 57%, and 35% [5]. Despite advances in the treatment strategies over the past 30 years, the prognosis of patients remains poor and the quality of life (QOL) remains impaired [6].

Thus, novel effective and safe treatment options for these patients are highly wanted. Chinese herbal medicine (CHM), especially combined herbal prescription and external treatment, is widely applied in traditional Chinese medicine (TCM) to treat CHF, and in recent years, more and more randomized controlled trials (RCTs) have confirmed its advantages in CHF treatment [7–9]. The Chinese herbal prescription, Qishen Taohong granules (QTGs), is composed of nine herbal drugs that can nourish Qi, disperse blood stasis, and remove water. In our previous studies, QTGs had a good effect in improving the cardiac function while enhancing the QOL of CHF patients [10, 11]. The positive role of TCM external treatment on CHF had also been confirmed [7]. We propose that CHF patients treated with QTG combined with TCM external treatment will have more positive clinical outcomes than patients receiving the control drug.

However, to the best of our knowledge, reliable evidence-based medical data have not yet been reported for the treatment of CHF with TCM comprehensive interventions (CHM combined with external treatment). Within this context, a placebo-controlled, double-blind, and randomized crossover trial will be carried out to assess the efficacy of QTG combined with TCM external treatment in treating CHF.

## 2. Methods and Analysis

### 2.1. Design

**Figure 1:**
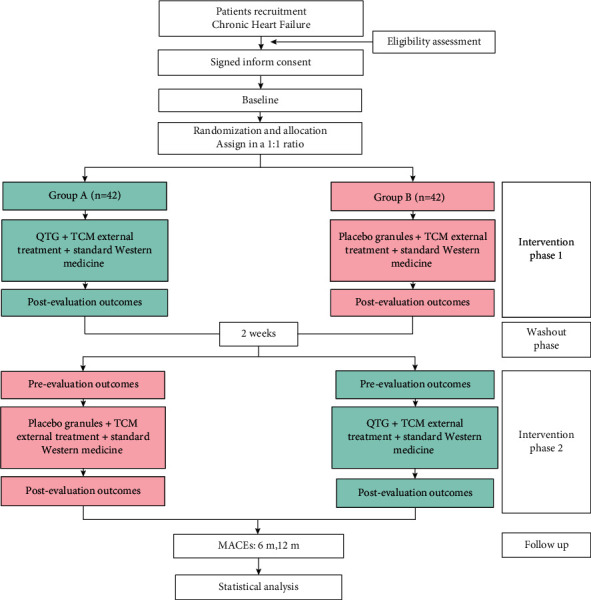
Study flow diagram.

This is a single-center, prospective, randomized, placebo-controlled, double-blind, crossover trial. Also, this trial has been formally enrolled at https://www.chictr.org.cn (ChiCTR2000038737). This study protocol promised the ethical requirements according to the 1975 Declaration of Helsinki. Meanwhile, this research has been permitted by our institutional review broad (JDF-IRB-2020031002), and the study flow is shown in Figure 1.

### 2.2. Participants

#### 2.2.1. Diagnostic Criteria

In order to ensure the scientific validity of the study, Framingham heart failure criteria are employed to diagnose CHF [12]. The reference of cardiac function staging originates from the American Heart Association (AHA) CHF staging [13]. The base of cardiac function grading lies in the grading protocol established by the former association NYHA [14]. TCM syndrome is identified by the treatment of heart failure as it writes “New drug investigation research guidelines for traditional Chinese medicine [15]”.

#### 2.2.2. Inclusion Criteria

The qualified subjects of this trial need to fulfill the following standards: (1) age between 18 and 75 years, (2) men and women who have been diagnosed as CHF, on the stage C of NYHA grade II or III, (3) TCM syndrome in forms of differentiation Qi deficiency, blood stasis, and water retention, (4) left ventricular ejection fraction (LVEF) < 40% or 40% ≤ LVEF ≤ 50% and NT-proBNP > 300 ng/L, and (5) provision of written informed consent.

#### 2.2.3. Exclusion Criteria

Subjects will be excluded if they had (1) acute myocardial infarction, (2) difficulties with walking, (3) severe mental dysfunction, pregnancy, or any malignancy, (4) severe liver and/or renal dysfunction, (5) severe anemia, and (6) taken TCM (including Chinese patent medicine or TCM injection or Chinese herbal medicine) or participated in other trials within 2 weeks.

#### 2.2.4. Termination Criteria

The termination criteria are as follows: (1) those who do not meet the inclusion criteria but are mistakenly enrolled, (2) those who are failing to follow the study protocol, (3) those who are having serious drug allergies or adverse reactions, and (4) various other reasons such as the patients who were asked to withdraw before the trial ended.

### 2.3. Randomization and Masking

Subjects will be divided into group A and B by the ratio of 1 : 1 by using sequentially numbered, sealed, opaque envelopes. The grouping and sequence will be summarized and stored on a computer by an independent statistician with no knowledge of the study design.

### 2.4. Blinding

Double blinding will be adopted in this study. Patients, investigators, outcome evaluators, and data analysts will all be unaware of treatment allocation throughout the study period. In terms of appearance, texture, color, and taste, placebo granules and QTGs are quite similar. At the end of this study, the emergency envelope will be assigned for every drug number, which represented the group information of the drug. The envelopes could only be opened when medical emergencies occur. In addition, once the emergency envelope is opened, the operator will be asked to sign and enter the date in the margins on the cover of letters, as well as the reasons of opening. After all the research data had been collected and recorded, the unblinding process will be carried out.

### 2.5. Intervention

Group A subjects will be given oral QTG combined with TCM external treatment and standard Western medicine for 4 weeks. Following a 2-week washout, patients in group A will input oral placebo granules combined with TCM external treatment and standard Western medicine for 4 weeks. Group B patients will be involved in the opposite medical procedure. During the washout period, patients will only receive standard Western medicine.

#### 2.5.1. Standard Western Medicine

The standard Western medicine follows the guidelines for CHF treatment [16]: (1) angiotensin-converting enzyme inhibitor (ACEI) or angiotensin II receptor blocker (ARB) or angiotensin receptor neprilysin inhibitors (ARNIs): fosinopril 10 mg once a day or valsartan capsule 80 mg once a day or other similar drugs, (2) beta-blockers (*β*-B): metoprolol succinate 47.5–190 mg once a day or bisoprolol fumarate 5–10 mg once a day, (3) diuretics: hydrochlorothiazide 12.5–25 mg once a day or furosemide 20 mg once or twice a day or tolasamide 20 mg once or twice a day (combined with 1 g potassium chloride sustained release tablets once to three times a day, depending on the patient's condition), (4) isosorbide dinitrate: aldosterone receptor antagonist: spironolactone 20 mg once a day, and (5) digoxin once a day if necessary.

#### 2.5.2. TCM External Treatment

(*1) Acupoint Sticking Therapy* [17]. The acupoint sticking therapy mainly consisted of *Salvia miltiorrhiza* 10 g, cassia twig 10 g, *Ligusticum wallichii* 10 g, and *Pericarpium trichosanthis* 10 g, and the ointment of acupoint sticking will be applied with patching time 4 h of each and once every three days. The acupuncture points will be selected are *shenque*, *mingmen,* and *danzhong*. Acupoint application for all patients will be performed by the same trained nurse.

*(2) Tongue Exercise* [18]. The patient will do the following steps once a day: licking the upper jaw 30 times, licking cheeks 4 times, licking gums 8 times, licking the corners of the mouth 10 times, slapping the tongue 20 times, and stretching the tongue 10 times. They would also be required to complete the tongue exercise, under the guidance of the same trained nurse.

*(3) Auricular Point Acupressure* [19]. The specific ear points that will be treated are the heart, shenmen and sympathesis, chest, and other points. After ear skin was disinfected with alcohol cotton balls, 0.5 cm × 0.5 cm square tapes with the seeds of cowherb will be pressed on the established ear points until the patients experience a sense of acid distention (de *qi*), 5 times a day, with 2 min time of each. The seeds of cowherb will be replaced weekly. The same well-trained nurse performed all auricular point acupressure.

#### 2.5.3. QTGs

QTGs were obtained from Beijing Kangrentang Pharmaceutical Co., Ltd. (Beijing, China). The composition of the prescription is as follows: 60 grams (g) of *Astragalus membranaceus*, 15 g of *Codonopsis pilosula*, 15 g of *Salvia miltiorrhiza*, 10 g of *Semen persicae*, 10 g of *Carthamus tinctorius*, 10 g of *Cortex mori*, 15 g of *Semen lepidii*, 15 g of *Polyporus umbellatus*, and 15 g of *Lycopus lucidus*. Patients in phase 1 of group A and phase 2 of group B will receive QTG 11.2 g (brewed with 100–200 ml water) twice a day for 4 weeks.

#### 2.5.4. Placebo

The placebo of QTGs that was made up of dextrin, a bitter agent, and 5% dose of the QTG composition was also prepared by the same company. It is similar to the QTGs in terms of appearance, taste, and smell. Patients in phase 2 of group A and phase 1 of group B will receive placebo granules 11.2 g (brewed with 100–200 ml water) twice a day.

### 2.6. Outcomes

#### 2.6.1. Primary Outcome

The primary outcome is NYHA classification [15]. The NYHA classification is increased by at least 2 to be markedly effective, increased by 1 to be effective, increased by less than 1 to be ineffective, and reduced by at least 1 to be deteriorating.

#### 2.6.2. Secondary Outcomes

The following secondary outcomes will be marked before treatment and after treatment: (1) echocardiogram, (2) N-terminal pro-B-type natriuretic peptide (NT-proBNP), (3) Chronic Heart Failure Quality of Life Scale of Integrated Chinese and Western Medicine (CHFQLS) [20], (4) TCM syndrome score, (5) symptom score, (6) sign score, (7) six-minute walk test (6MWT), (8) Pittsburgh Sleep Quality Index (PSQI), (9) Montreal cognitive assessment (MoCA), (10) major adverse cardiovascular events (MACEs), and (11) metabolomics.

#### 2.6.3. Safety Outcomes

The safety assessment is based on vital signs, laboratory tests, and adverse reactions (ARs). Vital signs could be observed by breathing, blood pressure, heart rate, and temperature. Laboratory tests are mainly blood routine, renal function, liver function, urine routine, and serum electrolytes. The research team will specifically record the abnormal events during the study period.

If serious ARs occur, the researcher should immediately report to the ethics committee and take timely treatment measures. The schedule of intervention and assessments is shown in Table 1.

### 2.7. Sample Size Estimation

Estimating the effective rate according to the classification of NYHA, the sample size has been figured out. Based on previous study results, the effective rate in the QTG group was 84%, while the effective rate of the control group is around 60% [11]. According to the formula *n*1 = *n*2 = 2‾p‾q (*Z*_*α*_ + *Z*_*β*_)^2^/(p1 − p2)^2^ = 74, the result is *n* = 2 × 74 = 148 subjects. Also, the sample size for the crossover intervention trial is generally half that of the randomized controlled trial under the same situation [22]. Moreover, because the dropout rate is about 10%, 82 subjects are supposed to be randomly accounted to ensure the prescribed number of patients.

### 2.8. Data Collection and Management

The case record form (CRF) will be applied to the collection of relevant medical records. The CRF will be reviewed by clinical investigators and supervisors to be checked. Data were recorded in MS Excel 2007. For the data accuracy, two administrators will intervene and proofread independently. After the review and confirmation, the database will be locked. In principle, the locked data file cannot be modified again. If errors will be found, they will be corrected in the process of statistical analysis after confirmed, recorded, and explained.

### 2.9. Statistical Analysis

Intention-to-treat (ITT) and per-protocol (PP) analysis would be planned while the same results indicate that the evaluation results are reliable. All the randomized data would be analyzed by the ITT analysis, and missed data due to withdrawal will be finally substituted by the last observation carried forward (LOCF) or by the intragroup mean of the variable. If necessary, the missing value processing method provided by SPSS software will be adopted. The PP dataset will be used to analyze patients with good adherence to the research protocol.

Continuous data are represented by mean ± standard deviation (SD), and categorical data are represented by the degree of frequency. Considering the normal distributed continuous variable items, the independent *t*-test will be used for comparison between group A and group B, and the paired *t*-test will be used for comparison within each group; otherwise, a nonparametric test will be taken into consideration. Categorical variables will be analyzed with the help of Wilcoxon's test and the chi-squared test. For the correlation analysis of the two continuous data, when the data meet normality, Pearson correlation analysis will be used; otherwise, Spearman rank correlation analysis will be used.

*P* < 0.05 means that there is huge difference in the statistics. All data will be analyzed using the Statistical Product and Service Solutions (SPSS) unless otherwise noted.

### 2.10. Monitoring

Monitoring will be conducted by two members of the clinical research centre independent of the study group. Study visits will occur throughout the conduct of the study at least once each year. When nonconcordance occurred, a report will be immediately given to the principal investigator, and a second visit will be scheduled.

## 3. Discussion

Our study is the first double-blinded, randomized, placebo-controlled trial to investigate whether the TCM comprehensive intervention (QTGs combined with TCM external treatment) has a greater effect in patients with CHF.

CHF is a complex condition, with high morbidity, mortality, and treatment costs [23]. The current treatment of CHF has limitations [24]. Complementary and alternative therapies are, therefore, required in some patients with CHF. Evidence about the effectiveness of TCM comprehensive interventions for CHF patients is also limited. The study results are poorly comparable and have little scientific proof, and it is difficult to adequately illustrate the advantages of TCM.

Several clinical studies have shown that TCM improves the clinical symptoms, cardiac function, and QOL in people with CHF [25–27]. However, there are still few studies on the high level of evidence in this field, and most of them are based on Chinese herbal medicine. In the meantime, tongue exercise, acupoint sticking therapy, auricular point acupressure, and other TCM external treatments are also playing a significant role in clinics. It also fully embodies the characteristics of being simple, convenient, cheap, and effective in TCM, which is easy to master and popularize. The TCM comprehensive intervention can break through the limitation of the current TCM treatment program for CHF by using only prescriptions so that a large number of CHF patients distributed in Western medicine hospitals and primary hospitals can also benefit from it, and it has broad application prospects. Therefore, TCM comprehensive intervention is a potential feasible candidate for the CHF treatment.

QTGs are a TCM prescription with good clinical effects. Our previous studies have shown that QTGs could enhance CHF patients' cardiac function, TCM syndrome score, and QOL of patients with CHF [10, 11]. They also display a cardioprotective effect in animal models of heart failure and prevent left ventricular remodeling [28]. However, the effect of QTGs combined with TCM external treatment on CHF still requires confirmation.

Our study has strengths to a certain degree. Compared to the previous study, it is the first double-blind, placebo-controlled crossover study to assess the validity as well as the safety of TCM comprehensive intervention in CHF treatment. A crossover trial is a good choice in clinical studies because it avoids differences between groups and eliminates differences between individuals, thereby improving the accuracy of efficacy evaluation by evaluating the results in different medical interventions for each patient [29]. Also, the required sample size of a crossover trial is smaller than that of an RCT, which better solves the problem of limited number of subjects and improves the evidence-based medicine evidence for the treatment of CHF with TCM comprehensive intervention [30]. These will make up the shortcomings of the previous studies and make potential results generalizable.

However, this study also has some limitations. First, it lacks new oral drugs (such as Sacubitril-Valsartan and SGLT-2i) for the treatment of CHF as a positive drug. In addition, this study will be performed in China, and it is uncertain whether other ethnic groups and regions can achieve similar effects.

Thus, due to the lack of similar research, we believe that the performance of our study and publication of the results will provide scientific support for the use of TCM comprehensive intervention in individuals with CHF to improve their cardiac function and QOL.

## Figures and Tables

**Table 1:** Schedule of intervention and assessments.

Study Period														
	Enrolment	Allocation	After allocation											
			Intervention phase 1				Washout		Intervention phase 2				Follow-up	
Time point	Wk 1	Wk 0	Wk 1	Wk 2	Wk 3	Wk 4	Wk 5	Wk 6	Wk 7	Wk 8	Wk 9	Wk 10	6 mo	12 mo
Enrolment														
Eligibility screen	×													
Informed consent	×													
Randomization		×												
Interventions														
Group A			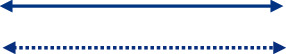				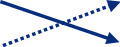		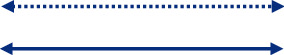					
Group B														
AssessmentsPrimary outcome														
NYHA classification		×				×		×				×		
Secondary outcomes														
Echocardiogram		×				×		×				×		
NT-proBNP		×				×		×				×		
CHFQLS		×				×		×				×		
TCM syndrome score		×				×		×				×		
Symptom score		×				×		×				×		
Sign score		×				×		×				×		
6MWT		×				×		×				×		
PSQI		×				×		×				×		
MoCA		×				×		×				×		
MACEs		×				×		×				×	×	×
Metabolomics		×				×								
Safety outcomes														
Liver and kidney function		×				×		×				×		
ARs						×		×				×		

Schedule of enrollment, intervention, and assessment for the MIRACLE trial was according to the Standard Protocol Items: Recommendations for Interventional Trials (SPIRIT) [21]. Wk, week; mo, month.

## Data Availability

The study is currently in the stage of recruitment of participants. The results of this study along with the full intervention protocol and participants' data will be published upon completion.

## Abbreviations

CHF: Chronic heart failure
TCM: Traditional Chinese medicine
QTGs: Qishen taohong granules
NYHA: New York Heart Association
NT-proBNP: N-terminal pro-B-type natriuretic peptide
CHFQLS: Chronic Heart Failure Quality of Life Scale of Integrated Chinese and Western Medicine
MoCA: Montreal cognitive assessment
MACEs: Major adverse cardiovascular events
QOL: Quality of life
CHM: Chinese herbal medicine
RCTs: Randomized controlled trials
AHA: American Heart Association
ACEI: Angiotensin-converting enzyme inhibitor
ARB: Angiotensin II receptor blocker
ARNI: Angiotensin receptor neprilysin inhibitors
*β*-B: Beta-blockers
LVEF: Left ventricular ejection fraction
6MWT: Six-minute walk test
PSQI: Pittsburgh Sleep Quality Index
Wk: Week
mo: Month
ARs: Adverse reactions
CRF: Case record form
ITT: Intention-to-treat
PP: Per-protocol
LOCF: Last observation carried forward
SD: Standard deviation
SPSS: Statistical Product and Service Solutions.

## Supplementary Materials

Supplementary Materials Additional file 1: Completed Standard Protocol Items: Recommendation for Interventional Trials (SPIRIT) 2013 Checklist: items addressed in this clinical trial protocol. Additional file 2: informed consent form.
