# Supplementary material for: A Single-Central, Randomized, Double-Blinded, Placebo-Controlled, Crossover Trial Protocol: A Clinical Effect Evaluation Study on the TCM Comprehensive Intervention Program for Chronic Heart Failure
Source: Evid Based Complement Alternat Med. 2021 Dec 28;2021:4577139. doi: 10.1155/2021/4577139 (PMC11401715; doi:10.1155/2021/4577139)
Supplement: Supplementary Materials — Additional file 1: Completed Standard Protocol Items: Recommendation for Interventional Trials (SPIRIT) 2013 Checklist: items addressed in this clinical trial protocol. Additional file 2: informed consent form. [file 4577139.f1.zip › 4577139.f1/Additional file 2.docx]

**Informed Consent Form·Notification Page**

Dear subjects:

We invite you to participate in the research project of the Capital Health Development Scientific Research Project "Clinical study on TCM Comprehensive intervention program and curative effect evaluation of chronic heart failure based on Yiqi Huoxue Lishui method". It is estimated that 80 subjects will participate voluntarily in this study. This study has been reviewed and approved by the Ethics Committee of Dongfang Hospital.

**1.Why do you want to carry out this research?** Chronic heart failure is a clinical syndrome in which ventricular filling or ejection fraction is reduced due to abnormalities in the structure or function of the heart. It is the final stage of the development of various cardiovascular diseases. Although the basic research of heart failure has made many remarkable achievements in recent years, the application of various new drugs and heart assist devices has also prolonged the survival time and quality of life of many heart failure patients. A large number of patients with heart failure still face the problems of low quality of life, repeated relapses, and toxic side effects, especially in terms of quality of life. Chinese medicine has achieved fruitful results in the treatment of chronic heart failure. It has shown great advantages in enhancing efficacy, relieving symptoms, prolonging life, avoiding adverse reactions, etc., especially in improving the quality of life of patients, attracting more and more Western medical colleagues. This topic uses our hospital’s proven prescription for the treatment of heart failure—Qishen Taohong Granule, as well as comprehensive traditional Chinese medicine treatments to observe the improvement of chronic heart failure patients’ quality of life, heart function, cardiac ultrasound, 6MWT, NT-proBNP and other indicators. To provide more clinical evidence for TCM treatment of heart failure.

**2. What is the subject population of this study?** The inclusion criteria of the subjects in this study were to sign an informed consent form, in line with the American College of Cardiology and the American Heart Association (ACC/AHA) chronic heart failure stage B/C, and the cardiac function grading Ⅱ-Ⅲ mild to moderate Patients with chronic heart failure, aged between 18 and 80 years old.

**3.Who should not participate in the study?** Patients with acute myocardial infarction, pregnancy, psychosis, malignant tumors, dementia or mental disorders, allergic constitutions and allergies to multiple drugs, severe liver and kidney dysfunction, and severe anemia will not be able to participate in this study.

**4. What do I need to do if I participate in study?** This study is a a randomized, double-blind, placebo-controlled, crossover trial. If you are willing to participate in this study, we will perform the symptoms, signs, quality of life, heart function, cardiac ultrasound, 6MWT, echocardiography, NT-proBNP and other indicators when you are admitted to the hospital. You will have a 50% chance of entering the control group and receive basic western medicine treatment, placebo granules and external TCM treatment, and 50% chance of entering the treatment group, plus Qishen Taohong granules and traditional Chinese medicine treatment on the basis of western medicine treatment, The course of treatment is 4 weeks. After that, you will enter a two-week wash-out period, and in the next four weeks, you will enter another group to receive the corresponding treatment. We will follow up with you, and test the symptoms, signs, quality of life, heart function, echocardiography, 6MWT, echocardiography, NT-proBNP and other indicators in the 4th and 10th week respectively.

**5. What are the risks of participating in the research?** All treatment drugs may have adverse reactions. The western medicine used in the control group in this study protocol is the conventional treatment for clinical treatment of heart failure, and there may be adverse reactions such as electrolyte imbalance and gastrointestinal symptoms; the Qishen Taohong granule as a famous Chinese medicine experience prescription in our department. Long-term clinical application has shown that it has a significant effect on improving symptoms of heart failure, fatigue, shortness of breath, chest tightness, and facial and limb edema. No obvious adverse reactions occurred during the application.

Regardless of any discomfort or changes in your condition during the study, please contact your doctor in time and we will treat you in time. If the investigator gets information that may affect your continued participation in the trial, he will also notify you in time.

**6. What are the benefits of participating in research?** By participating in this study, your heart failure condition/symptoms may be improved. This study will also help determine which treatments can be safer and more effective to treat other patients with similar conditions.

**7. Do I need to pay for participating in the research?** In order to compensate you for the inconvenience that may be caused by your participation in this study, this study will pay for the cost of taking traditional Chinese medicine granular formula and the quality of life, heart function, cardiac ultrasound, 6MWT, ultrasound for 4 weeks and 10 weeks. Cardiopulmonary, NT-proBNP examination fees, other medication and examination fees during hospitalization are not included in the free range. If you combine treatments and examinations required for other diseases, as well as the cost of switching to other treatments due to ineffective treatments, it will not be free of charge.

During the research process, we will closely monitor the adverse reactions and take measures to prevent them. If research-related damage occurs, you will be entitled to corresponding compensation.

**8. Is personal information confidential?** Your medical records will be kept in the hospital, and investigators, research authorities, and ethics committees will be allowed to access your medical records. Any public report on the results of this research will not disclose your personal identity. We will make every effort to protect the privacy of your personal medical data within the bounds of the law.

**9. Do I have to participate in the study?** Whether to participate in the study is entirely up to your volition. You can refuse to participate in this study, or withdraw from this study at any time during the study process, this will not affect the relationship between you and the doctor.

If you do not participate in this study, or withdraw from the study halfway, you can still take Western medical treatment alone. You do not have to choose to participate in this study in order to treat your disease.

Your doctor or investigator may also suspend your participation in this study at any time for your best interests. If you withdraw from the study for any reason, you may be asked about your use of the trial drug. If the doctor thinks it is necessary, you may also be asked for laboratory tests and physical examinations. This is very beneficial to protect your health.

**10. Other**

If you have any medical questions related to this study, please contact Dr. Li Yan at +86 010 67689757.

If you want to reflect any dissatisfaction and worries during the process of participating in this research, or if your personal rights and interests have been harmed, you can contact the Clinical Research Ethics Committee of Oriental Hospital at +86 010 67654807.

**Informed Consent·Signature Page**

**Prompt before signing:** Please read the information carefully before signing the informed consent. You have the right to ask us for any questions about this study and its information. You should give enough time to fully and carefully consider, and you can discuss with family members and friends to help you make a decision whether to participate in the trial. This informed consent form is in duplicate, and you will keep a signed and dated copy of the informed consent form.

**Subject statement:** I have read the above introduction to this research and fully understand the possible risks and benefits of participating in this research. I voluntarily participate in this research.

Subject's signature: Date:

Subject's contact number: Phone number:

**Investigator statement:** I confirm that I have explained the details of this research to the subjects, especially the possible risks and benefits of participating in this research.

Investigator’s signature: Date:

Investigator’s contact number： Phone number:

**Informed Consent·Signature Page**

**Prompt before signing:** Please read the information carefully before signing the informed consent. You have the right to ask us for any questions about this study and its information. You should give enough time to fully and carefully consider, and you can discuss with family members and friends to help you make a decision whether to participate in the trial. This informed consent form is in duplicate, and you will keep a signed and dated copy of the informed consent form.

**Subject statement:** I have read the above introduction to this research and fully understand the possible risks and benefits of participating in this research. I voluntarily participate in this research.

Subject's signature: Date:

Subject's contact number: Phone number:

**Investigator statement:** I confirm that I have explained the details of this research to the subjects, especially the possible risks and benefits of participating in this research.

Investigator’s signature: Date:

Investigator’s contact number： Phone number:
